# Poly[tetra­aqua­bis(μ_2_-2,4,6-trinitro­phenolato)barium(II)]

**DOI:** 10.1107/S1600536808002961

**Published:** 2008-02-06

**Authors:** C. Vesta, R. Uthrakumar, S. Jerome Das, Babu Varghese

**Affiliations:** a Department of Physics, Loyola College, Chennai 600 034, India; b Sophisticated Analytical Instruments Facility, Indian Institute of Technology Madras, Chennai 600 036, India

## Abstract

The asymmetric unit of the title compound, [Ba(C_6_H_2_N_3_O_7_)_2_(H_2_O)_4_]_*n*_, consists of a barium ion coordinated by two nitrophenolate ligands and four water mol­ecules. Barium is deca­coordinated by O atoms. These units are linked together through bridging nitro groups to form a one-dimensional polymeric chain. The three-dimensional packing is facilitated through hydrogen-bonding inter­actions mediated through water mol­ecules. The coordination distances around Ba vary from 2.728 (4) to 3.138 (5) Å. The crystal sample, on exposure to air at room temperature for many days, slowly loses the water and peels out as filaments.

## Related literature

For related literature, see: Brahadeeswaran *et al.* (1998 ▶, 1999 ▶); Jonie Varjula *et al*. (2007 ▶); Milton Boaz *et al*. (2005 ▶); Vesta *et al.* (2007 ▶).
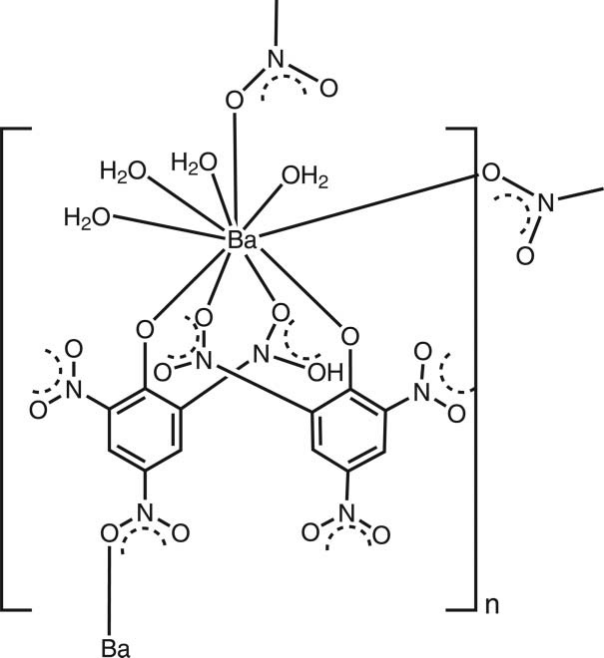


## Experimental

### 

#### Crystal data

[Ba(C_6_H_2_N_3_O_7_)_2_(H_2_O)_4_]
*M* *_r_* = 665.62
Monoclinic, 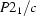

*a* = 11.6765 (4) Å
*b* = 6.6878 (2) Å
*c* = 27.0324 (9) Å
β = 95.608 (2)°
*V* = 2100.86 (12) Å^3^
*Z* = 4
Mo *K*α radiation
μ = 2.00 mm^−1^
*T* = 293 (2) K
0.20 × 0.20 × 0.15 mm

#### Data collection

Bruker Kappa APEX2 diffractometer
Absorption correction: multi-scan (*SADABS*; Bruker, 1999 ▶) *T*_min_ = 0.652, *T*_max_ = 0.723
20976 measured reflections
3674 independent reflections
3552 reflections with *I* > 2σ(*I*)
*R*_int_ = 0.020

#### Refinement

*R*[*F* ^2^ > 2σ(*F* ^2^)] = 0.044
*wR*(*F* ^2^) = 0.084
*S* = 1.42
3674 reflections
360 parameters
234 restraints
H atoms treated by a mixture of independent and constrained refinement
Δρ_max_ = 0.58 e Å^−3^
Δρ_min_ = −0.53 e Å^−3^

### 

Data collection: *APEX2* (Bruker, 2004 ▶); cell refinement: *APEX2* and *SAINT* (Bruker, 2004 ▶); data reduction: *SAINT* and *XPREP* (Bruker, 2004 ▶); program(s) used to solve structure: *SIR92* (Altomare *et al.*, 1993 ▶); program(s) used to refine structure: *SHELXL97* (Sheldrick, 2008 ▶); molecular graphics: *ORTEP-3* (Farrugia, 1997 ▶) and *Mercury* (Macrae *et al.*, 2006 ▶); software used to prepare material for publication: *SHELXL97*.

## Supplementary Material

Crystal structure: contains datablocks global, I. DOI: 10.1107/S1600536808002961/sg2220sup1.cif

Structure factors: contains datablocks I. DOI: 10.1107/S1600536808002961/sg2220Isup2.hkl

Additional supplementary materials:  crystallographic information; 3D view; checkCIF report

## Figures and Tables

**Table 1:** Hydrogen-bond geometry (Å, °)

*D*—H⋯*A*	*D*—H	H⋯*A*	*D*⋯*A*	*D*—H⋯*A*
O15—H15*A*⋯O7^i^	0.85 (4)	2.20 (4)	2.939 (6)	145 (6)
O15—H15*A*⋯O6^i^	0.85 (4)	2.33 (4)	3.030 (7)	140 (5)
O15—H15*B*⋯O9^ii^	0.85 (4)	2.103 (6)	2.953 (6)	179 (7)
O16—H16*A*⋯O4^iii^	0.85 (3)	2.08 (4)	2.806 (6)	142 (5)
O16—H16*B*⋯O15^iv^	0.85 (6)	2.11 (3)	2.906 (7)	156 (6)
O17—H17*A*⋯O12^v^	0.85 (4)	2.45 (3)	3.258 (8)	158 (6)
O17—H17*B*⋯O18^v^	0.85 (5)	1.969 (17)	2.805 (7)	168 (7)
O18—H18*A*⋯O16^i^	0.85 (4)	2.04 (3)	2.822 (7)	153 (7)
O18—H18*B*⋯O1^vi^	0.85 (5)	2.47 (3)	3.241 (7)	151 (6)

Symmetry codes: (i) 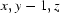; (ii) 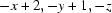; (iii) 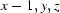; (iv) 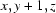; (v) 
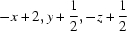
; (vi) 
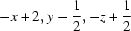
.

**Fig. 1.**
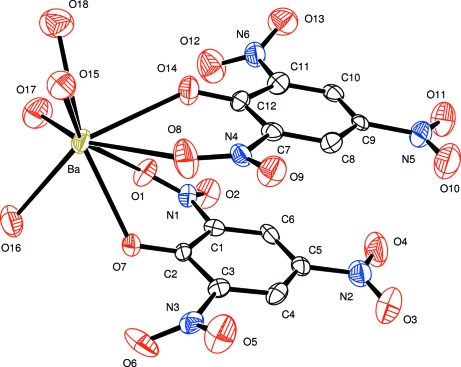
The ORTEP representation of the molecule with 50% probability anisotropic ellipsoid.

**Fig. 2.**
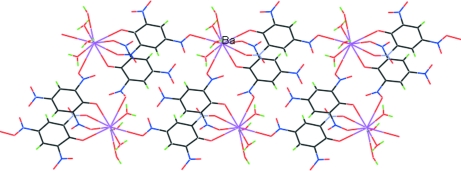
One dimensional polymeric chain of the title compound formed by a-translation of the asymmetric unit and its inversion.

**Fig. 3.**
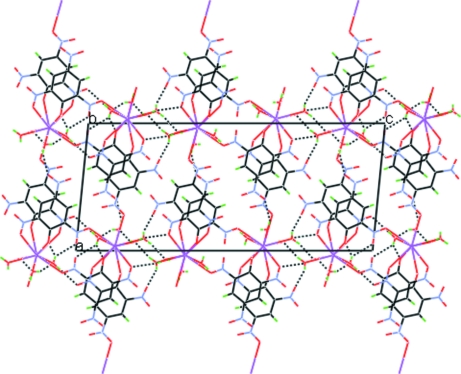
Packing of molecules in the unit cell viewed down b axis. Hydrogen bonds are shown with dotted lines.

## supplementary crystallographic information

### Comment

Nitrophenol family of crystals are found to have high laser damage threshold, wide transparency windows, and high NLO co-efficients ((Brahadeeswaran *et al.*, 1998, 1999), (Milton Boaz *et al.*, 2005), (Vesta *et al.*, 2007), (Jonie Varjula *et al.*, 2007)). Nitrophenol groups are found to be good proton acceptors from the metallic hydroxide complexes. The title compound was synthesized as part of our ongoing research for synthesizing and characterizing new optically active materials. In the present work, the crystal structure of the compound (BaC_12_H_4_N_6_O_14_.4H_2_O) is reported for the first time.The reported compound is not optically active.

The title compound crystallizes in monoclinic system with space group *P*2_1_/*c*. *ORTEP* representation of the molecule with 50% anisotropic ellipsoids are shown in figure1.The asymmetric unit consists of two nitrophenolate moieties coordinated to barium through phenolate O atoms O7 and O14 and one nitro oxygen each from nitrophenolate moieties (O1 and O8), on one side. Four water molecules of the asymmetric unit coordinates to other side. The asymmetric unit and its inversion are linked to each other through nitro oxygen O5(symm: 2 - *x*, 2 - *y*, -*z*) coordinating to metal. The centrosymmetric pair and its a-translations are joined to each other through nitro O atoms O11 (symm: *x* - 1, *y*, *z*) to form an one dimensional infinite polymeric chain parallel to *a* axis (Fig.2).Thus, Barium is coordinated with 10 O atoms. The coordination distances around Ba vary from 2.728 Å to 3.138 Å. The one dimensional chains are further linked to each other (along b and c directions) through water mediated O—H···O hydrogen bonds (Fig.3). The crystal sample, on exposure to air at room temperature for many days, slowly looses the water and peels out as filaments.

### Experimental

Picric acid (99%, 5.73 g ms) was dissolved in deionized water (100 ml) and then Ba(OH)2 (97%, 3.94 g ms) was added slowly with stirring to obtain saturated solution. The saturated solution kept at 305 K yielded fine yellow crystals in three days through spontaneous nucleation. The sample was purified further through recrystallization.

### Refinement

The aromatic H atoms were located in Fourier difference map and geometrically constrained at idealized positions (C—H = 0.93 Å) and were given riding model refinement with *U*_iso_ equal to 1.2 times *U*_eq_ of the parent carbon. All the water H atoms were located in difference Fourier map and refined isotropically with following restrints: O—H = 0.850 (1)Å and H···H = 1.380 (1) Å. These restraints were put to avoid bad geometry after refinement. The isotropic thermal parameters of H atoms H16A, H16B, H17A, H17B, H18A and H18B were constrained as 0.08 Å^-2^ during refinement.

### Crystal data

**Table:**

[Ba(C_6_H_2_N_3_O_7_)_2_(H_2_O)_4_]	*F*_000_ = 1304
*M**_r_* = 665.62	*D*_x_ = 2.104 Mg m^−^^3^
Monoclinic, *P*2_1_/*c*	Mo *K*α radiation λ = 0.71073 Å
Hall symbol: -P2ybc	Cell parameters from 6588 reflections
*a* = 11.6765 (4) Å	θ = 2.4–25.0º
*b* = 6.6878 (2) Å	µ = 2.00 mm^−^^1^
*c* = 27.0324 (9) Å	*T* = 293 (2) K
β = 95.608 (2)º	Plate, yellow
*V* = 2100.86 (12) Å^3^	0.20 × 0.20 × 0.15 mm
*Z* = 4	

### Data collection

**Table:**

Bruker Kappa APEX2 diffractometer	3674 independent reflections
Radiation source: fine-focus sealed tube	3552 reflections with *I* > 2σ(*I*)
Monochromator: graphite	*R*_int_ = 0.020
*T* = 293(2) K	θ_max_ = 25.0º
ω and φ scans	θ_min_ = 2.4º
Absorption correction: multi-scan(SADABS; Bruker, 1999)	*h* = −13→13
*T*_min_ = 0.652, *T*_max_ = 0.723	*k* = −7→7
20976 measured reflections	*l* = −32→32

### Refinement

**Table:**

Refinement on *F*^2^	Secondary atom site location: difference Fourier map
Least-squares matrix: full	Hydrogen site location: inferred from neighbouring sites
*R*[*F*^2^ > 2σ(*F*^2^)] = 0.044	H atoms treated by a mixture of independent and constrained refinement
*wR*(*F*^2^) = 0.084	*w* = 1/[σ^2^(*F*_o_^2^) + 9.4089*P*] where *P* = (*F*_o_^2^ + 2*F*_c_^2^)/3
*S* = 1.42	(Δ/σ)_max_ = 0.002
3674 reflections	Δρ_max_ = 0.58 e Å^−^^3^
360 parameters	Δρ_min_ = −0.53 e Å^−^^3^
234 restraints	Extinction correction: none
Primary atom site location: structure-invariant direct methods	

### Special details

**Table:**

Geometry. All e.s.d.'s (except the e.s.d. in the dihedral angle between two l.s. planes) are estimated using the full covariance matrix. The cell e.s.d.'s are taken into account individually in the estimation of e.s.d.'s in distances, angles and torsion angles; correlations between e.s.d.'s in cell parameters are only used when they are defined by crystal symmetry. An approximate (isotropic) treatment of cell e.s.d.'s is used for estimating e.s.d.'s involving l.s. planes.
Refinement. Refinement of *F*^2^ against ALL reflections. The weighted *R*-factor *wR* and goodness of fit *S* are based on *F*^2^, conventional *R*-factors *R* are based on *F*, with *F* set to zero for negative *F*^2^. The threshold expression of *F*^2^ > σ(*F*^2^) is used only for calculating *R*-factors(gt) *etc*. and is not relevant to the choice of reflections for refinement. *R*-factors based on *F*^2^ are statistically about twice as large as those based on *F*, and *R*- factors based on ALL data will be even larger.

### Fractional atomic coordinates and isotropic or equivalent isotropic displacement parameters (Å^2^)

**Table:**

	*x*	*y*	*z*	*U*_iso_*/*U*_eq_	
C1	1.2555 (4)	1.1054 (8)	0.13511 (18)	0.0242 (11)	
C2	1.1690 (4)	1.1014 (7)	0.09309 (19)	0.0238 (10)	
C3	1.2195 (4)	1.1225 (7)	0.04666 (18)	0.0255 (11)	
C4	1.3344 (4)	1.1314 (8)	0.04212 (19)	0.0280 (11)	
H4	1.3617	1.1369	0.0110	0.034*	
C5	1.4101 (4)	1.1319 (8)	0.0851 (2)	0.0271 (11)	
C6	1.3720 (4)	1.1214 (7)	0.13149 (19)	0.0258 (11)	
H6	1.4238	1.1251	0.1599	0.031*	
C7	1.3226 (4)	0.6198 (7)	0.09934 (18)	0.0246 (11)	
C8	1.4352 (4)	0.6309 (8)	0.0885 (2)	0.0280 (12)	
H8	1.4527	0.6363	0.0557	0.034*	
C9	1.5217 (4)	0.6338 (8)	0.12707 (19)	0.0268 (11)	
C10	1.4971 (4)	0.6212 (8)	0.1761 (2)	0.0301 (12)	
H10	1.5561	0.6187	0.2018	0.036*	
C11	1.3855 (5)	0.6125 (8)	0.18597 (19)	0.0291 (12)	
C12	1.2861 (4)	0.6108 (8)	0.1489 (2)	0.0270 (11)	
N1	1.2203 (4)	1.0954 (7)	0.18515 (16)	0.0324 (10)	
N2	1.5322 (4)	1.1492 (8)	0.0809 (2)	0.0409 (12)	
N3	1.1438 (4)	1.1353 (7)	0.00065 (17)	0.0339 (11)	
N4	1.2374 (4)	0.6143 (7)	0.05641 (16)	0.0284 (10)	
N5	1.6402 (4)	0.6485 (8)	0.11597 (19)	0.0384 (12)	
N6	1.3656 (4)	0.6031 (9)	0.23850 (18)	0.0426 (12)	
O1	1.1282 (4)	1.0179 (7)	0.19220 (15)	0.0450 (11)	
O2	1.2845 (4)	1.1681 (8)	0.21915 (15)	0.0524 (12)	
O3	1.5656 (4)	1.1695 (9)	0.03987 (19)	0.0671 (15)	
O4	1.5960 (4)	1.1459 (10)	0.1193 (2)	0.0709 (16)	
O5	1.1760 (4)	1.0643 (9)	−0.03692 (16)	0.0604 (14)	
O6	1.0527 (4)	1.2218 (8)	0.00097 (17)	0.0616 (14)	
O7	1.0641 (3)	1.0833 (6)	0.09589 (14)	0.0359 (9)	
O8	1.1364 (3)	0.6458 (8)	0.06148 (16)	0.0566 (13)	
O9	1.2699 (4)	0.5776 (7)	0.01567 (14)	0.0467 (11)	
O10	1.6600 (4)	0.6531 (9)	0.07262 (19)	0.0672 (15)	
O11	1.7160 (3)	0.6578 (8)	0.15020 (18)	0.0573 (13)	
O12	1.2822 (5)	0.6819 (10)	0.25207 (18)	0.0770 (17)	
O13	1.4367 (4)	0.5156 (8)	0.26667 (17)	0.0616 (14)	
O14	1.1854 (3)	0.5971 (7)	0.15895 (15)	0.0419 (10)	
O15	0.9006 (4)	0.4119 (7)	0.07251 (15)	0.0403 (10)	
O16	0.8349 (4)	1.1003 (8)	0.13919 (18)	0.0522 (12)	
O17	0.9062 (4)	0.8444 (9)	0.22439 (17)	0.0601 (13)	
O18	0.9301 (5)	0.4269 (8)	0.19520 (18)	0.0639 (14)	
Ba	0.96482 (2)	0.74829 (6)	0.130390 (11)	0.02924 (11)	
H15A	0.955 (3)	0.334 (7)	0.067 (2)	0.07 (3)*	
H15B	0.852 (4)	0.415 (11)	0.0470 (14)	0.08 (3)*	
H16A	0.7657 (17)	1.072 (11)	0.144 (3)	0.080*	
H16B	0.835 (5)	1.182 (10)	0.115 (2)	0.080*	
H17A	0.852 (3)	0.930 (6)	0.222 (2)	0.080*	
H17B	0.948 (5)	0.866 (11)	0.2514 (15)	0.080*	
H18A	0.925 (6)	0.318 (5)	0.179 (2)	0.080*	
H18B	0.901 (6)	0.410 (10)	0.2224 (15)	0.080*	

### Atomic displacement parameters (Å^2^)

**Table:**

	*U*^11^	*U*^22^	*U*^33^	*U*^12^	*U*^13^	*U*^23^
C1	0.030 (3)	0.020 (2)	0.023 (2)	0.002 (2)	0.003 (2)	0.002 (2)
C2	0.019 (2)	0.017 (2)	0.035 (3)	−0.002 (2)	0.003 (2)	0.001 (2)
C3	0.031 (3)	0.017 (3)	0.028 (3)	−0.004 (2)	0.000 (2)	0.001 (2)
C4	0.033 (3)	0.023 (3)	0.030 (3)	−0.004 (2)	0.010 (2)	−0.003 (2)
C5	0.023 (3)	0.021 (3)	0.038 (3)	0.002 (2)	0.004 (2)	−0.003 (2)
C6	0.024 (3)	0.019 (3)	0.033 (3)	0.003 (2)	−0.003 (2)	−0.002 (2)
C7	0.026 (3)	0.018 (2)	0.029 (3)	0.000 (2)	−0.004 (2)	0.000 (2)
C8	0.027 (3)	0.025 (3)	0.032 (3)	−0.001 (2)	0.006 (2)	0.002 (2)
C9	0.019 (2)	0.024 (3)	0.038 (3)	0.000 (2)	0.003 (2)	0.002 (2)
C10	0.026 (3)	0.027 (3)	0.036 (3)	−0.002 (2)	−0.007 (2)	0.003 (2)
C11	0.030 (3)	0.030 (3)	0.027 (3)	0.000 (2)	0.001 (2)	−0.001 (2)
C12	0.022 (3)	0.021 (3)	0.037 (3)	0.000 (2)	0.001 (2)	0.001 (2)
N1	0.031 (2)	0.036 (3)	0.030 (2)	−0.001 (2)	0.0047 (19)	−0.002 (2)
N2	0.028 (2)	0.042 (3)	0.054 (3)	0.001 (2)	0.008 (2)	−0.004 (2)
N3	0.033 (3)	0.036 (3)	0.032 (2)	−0.010 (2)	−0.0009 (19)	0.005 (2)
N4	0.027 (2)	0.023 (2)	0.034 (2)	0.0018 (19)	−0.0030 (18)	−0.0015 (19)
N5	0.023 (2)	0.041 (3)	0.051 (3)	−0.002 (2)	0.005 (2)	0.003 (2)
N6	0.035 (3)	0.054 (3)	0.039 (3)	−0.001 (3)	0.004 (2)	0.000 (3)
O1	0.040 (2)	0.058 (3)	0.039 (2)	−0.016 (2)	0.0174 (18)	−0.002 (2)
O2	0.050 (3)	0.075 (3)	0.032 (2)	−0.011 (2)	0.0019 (19)	−0.015 (2)
O3	0.042 (3)	0.095 (4)	0.069 (3)	−0.010 (3)	0.029 (2)	−0.006 (3)
O4	0.023 (2)	0.115 (4)	0.073 (3)	0.002 (3)	−0.004 (2)	0.008 (3)
O5	0.046 (3)	0.102 (4)	0.033 (2)	−0.012 (3)	0.0025 (19)	−0.013 (3)
O6	0.058 (3)	0.064 (3)	0.058 (3)	0.024 (3)	−0.019 (2)	0.002 (3)
O7	0.0214 (19)	0.039 (2)	0.047 (2)	−0.0024 (17)	0.0017 (16)	0.0081 (19)
O8	0.025 (2)	0.091 (4)	0.051 (3)	0.015 (2)	−0.0070 (18)	−0.014 (3)
O9	0.042 (2)	0.070 (3)	0.028 (2)	−0.006 (2)	0.0009 (17)	0.003 (2)
O10	0.040 (3)	0.106 (4)	0.059 (3)	0.001 (3)	0.019 (2)	0.013 (3)
O11	0.022 (2)	0.081 (3)	0.068 (3)	−0.007 (2)	−0.003 (2)	−0.003 (3)
O12	0.070 (3)	0.117 (5)	0.047 (3)	0.026 (3)	0.019 (2)	−0.006 (3)
O13	0.065 (3)	0.077 (4)	0.040 (2)	−0.001 (3)	−0.008 (2)	0.015 (2)
O14	0.024 (2)	0.055 (3)	0.047 (2)	0.0024 (19)	0.0071 (17)	0.003 (2)
O15	0.040 (2)	0.039 (2)	0.040 (2)	0.004 (2)	−0.0036 (19)	−0.0070 (19)
O16	0.033 (2)	0.054 (3)	0.069 (3)	0.006 (2)	0.004 (2)	−0.005 (2)
O17	0.060 (3)	0.084 (4)	0.038 (2)	−0.006 (3)	0.008 (2)	−0.001 (2)
O18	0.082 (4)	0.059 (3)	0.048 (3)	−0.010 (3)	−0.007 (3)	0.011 (2)
Ba	0.02237 (16)	0.03288 (18)	0.03255 (17)	−0.00217 (15)	0.00312 (11)	0.00155 (16)

### Geometric parameters (Å, °)

**Table:**

C1—C6	1.378 (7)	N3—O6	1.211 (6)
C1—C2	1.444 (7)	N3—O5	1.214 (6)
C1—N1	1.453 (6)	N4—O8	1.218 (6)
C2—O7	1.241 (6)	N4—O9	1.224 (6)
C2—C3	1.445 (7)	N5—O10	1.216 (6)
C3—C4	1.360 (7)	N5—O11	1.218 (6)
C3—N3	1.457 (7)	N6—O12	1.196 (7)
C4—C5	1.388 (7)	N6—O13	1.220 (7)
C4—H4	0.9300	O1—Ba	3.010 (4)
C5—C6	1.374 (7)	O5—Ba^i^	3.138 (5)
C5—N2	1.446 (7)	O7—Ba	2.728 (4)
C6—H6	0.9300	O8—Ba	2.947 (4)
C7—C8	1.377 (7)	O11—Ba^ii^	3.065 (4)
C7—C12	1.446 (7)	O14—Ba	2.805 (4)
C7—N4	1.453 (6)	O15—Ba	2.801 (4)
C8—C9	1.379 (7)	O15—H15A	0.85 (4)
C8—H8	0.9300	O15—H15B	0.85 (4)
C9—C10	1.385 (7)	O16—Ba	2.823 (5)
C9—N5	1.448 (6)	O16—H16A	0.85 (3)
C10—C11	1.356 (7)	O16—H16B	0.85 (6)
C10—H10	0.9300	O17—Ba	2.771 (4)
C11—C12	1.458 (7)	O17—H17A	0.85 (4)
C11—N6	1.463 (7)	O17—H17B	0.85 (5)
C12—O14	1.236 (6)	O18—Ba	2.827 (5)
N1—O1	1.225 (6)	O18—H18A	0.85 (4)
N1—O2	1.228 (6)	O18—H18B	0.85 (5)
N2—O3	1.218 (6)	Ba—O11^iii^	3.065 (4)
N2—O4	1.218 (7)	Ba—O5^i^	3.138 (5)
			
C6—C1—C2	124.4 (4)	C12—O14—Ba	141.3 (4)
C6—C1—N1	116.1 (4)	Ba—O15—H15A	116 (3)
C2—C1—N1	119.5 (4)	Ba—O15—H15B	124 (5)
O7—C2—C1	124.9 (5)	H15A—O15—H15B	109 (5)
O7—C2—C3	123.5 (5)	Ba—O16—H16A	111 (5)
C1—C2—C3	111.6 (4)	Ba—O16—H16B	116 (5)
C4—C3—C2	124.9 (5)	H16A—O16—H16B	109 (6)
C4—C3—N3	116.3 (5)	Ba—O17—H17A	109 (5)
C2—C3—N3	118.8 (4)	Ba—O17—H17B	131 (5)
C3—C4—C5	118.5 (5)	H17A—O17—H17B	108 (6)
C3—C4—H4	120.7	Ba—O18—H18A	110 (4)
C5—C4—H4	120.7	Ba—O18—H18B	137 (5)
C6—C5—C4	121.8 (5)	H18A—O18—H18B	109 (6)
C6—C5—N2	119.1 (5)	O7—Ba—O17	105.91 (15)
C4—C5—N2	119.1 (5)	O7—Ba—O15	124.63 (12)
C5—C6—C1	118.6 (5)	O17—Ba—O15	128.47 (15)
C5—C6—H6	120.7	O7—Ba—O14	88.90 (12)
C1—C6—H6	120.7	O17—Ba—O14	97.68 (14)
C8—C7—C12	125.0 (5)	O15—Ba—O14	93.02 (13)
C8—C7—N4	115.1 (4)	O7—Ba—O16	66.02 (13)
C12—C7—N4	119.9 (4)	O17—Ba—O16	63.03 (16)
C7—C8—C9	118.9 (5)	O15—Ba—O16	126.89 (13)
C7—C8—H8	120.5	O14—Ba—O16	139.82 (13)
C9—C8—H8	120.5	O7—Ba—O18	158.07 (13)
C8—C9—C10	121.1 (5)	O17—Ba—O18	62.93 (17)
C8—C9—N5	119.2 (5)	O15—Ba—O18	71.85 (14)
C10—C9—N5	119.6 (5)	O14—Ba—O18	74.86 (15)
C11—C10—C9	119.0 (5)	O16—Ba—O18	118.10 (16)
C11—C10—H10	120.5	O7—Ba—O8	68.72 (14)
C9—C10—H10	120.5	O17—Ba—O8	151.36 (13)
C10—C11—C12	125.5 (5)	O15—Ba—O8	67.76 (13)
C10—C11—N6	116.1 (5)	O14—Ba—O8	55.05 (12)
C12—C11—N6	118.4 (5)	O16—Ba—O8	130.63 (15)
O14—C12—C7	125.4 (5)	O18—Ba—O8	111.25 (17)
O14—C12—C11	124.0 (5)	O7—Ba—O1	55.28 (11)
C7—C12—C11	110.5 (4)	O17—Ba—O1	63.37 (13)
O1—N1—O2	122.1 (5)	O15—Ba—O1	155.78 (13)
O1—N1—C1	119.7 (4)	O14—Ba—O1	63.12 (13)
O2—N1—C1	118.1 (4)	O16—Ba—O1	76.72 (13)
O3—N2—O4	123.7 (5)	O18—Ba—O1	103.48 (13)
O3—N2—C5	119.1 (5)	O8—Ba—O1	93.27 (12)
O4—N2—C5	117.2 (5)	O7—Ba—O11^iii^	131.55 (13)
O6—N3—O5	122.5 (5)	O17—Ba—O11^iii^	64.06 (14)
O6—N3—C3	118.8 (5)	O15—Ba—O11^iii^	74.48 (13)
O5—N3—C3	118.6 (5)	O14—Ba—O11^iii^	137.85 (13)
O8—N4—O9	121.6 (4)	O16—Ba—O11^iii^	67.90 (14)
O8—N4—C7	120.0 (4)	O18—Ba—O11^iii^	62.99 (16)
O9—N4—C7	118.3 (4)	O8—Ba—O11^iii^	141.18 (13)
O10—N5—O11	122.6 (5)	O1—Ba—O11^iii^	125.55 (12)
O10—N5—C9	118.4 (5)	O7—Ba—O5^i^	66.63 (12)
O11—N5—C9	119.0 (5)	O17—Ba—O5^i^	119.27 (15)
O12—N6—O13	123.0 (6)	O15—Ba—O5^i^	77.31 (14)
O12—N6—C11	119.4 (5)	O14—Ba—O5^i^	139.51 (12)
O13—N6—C11	117.6 (5)	O16—Ba—O5^i^	59.42 (14)
N1—O1—Ba	133.3 (3)	O18—Ba—O5^i^	134.99 (15)
N3—O5—Ba^i^	109.7 (4)	O8—Ba—O5^i^	85.35 (12)
C2—O7—Ba	123.8 (3)	O1—Ba—O5^i^	117.49 (13)
N4—O8—Ba	146.8 (3)	O11^iii^—Ba—O5^i^	77.80 (13)
N5—O11—Ba^ii^	120.5 (4)		
			
C6—C1—C2—O7	178.6 (5)	C10—C11—N6—O12	146.4 (6)
N1—C1—C2—O7	−2.5 (8)	C12—C11—N6—O12	−33.9 (9)
C6—C1—C2—C3	−1.8 (7)	C10—C11—N6—O13	−32.8 (8)
N1—C1—C2—C3	177.2 (4)	C12—C11—N6—O13	146.9 (6)
O7—C2—C3—C4	−176.1 (5)	O2—N1—O1—Ba	−164.9 (4)
C1—C2—C3—C4	4.2 (7)	C1—N1—O1—Ba	15.9 (8)
O7—C2—C3—N3	3.8 (8)	O6—N3—O5—Ba^i^	17.7 (7)
C1—C2—C3—N3	−175.8 (4)	C3—N3—O5—Ba^i^	−160.0 (3)
C2—C3—C4—C5	−3.9 (8)	C1—C2—O7—Ba	−65.4 (6)
N3—C3—C4—C5	176.2 (5)	C3—C2—O7—Ba	115.0 (5)
C3—C4—C5—C6	0.8 (8)	O9—N4—O8—Ba	−178.2 (5)
C3—C4—C5—N2	−177.6 (5)	C7—N4—O8—Ba	1.9 (10)
C4—C5—C6—C1	1.5 (8)	O10—N5—O11—Ba^ii^	7.9 (8)
N2—C5—C6—C1	179.9 (5)	C9—N5—O11—Ba^ii^	−171.3 (4)
C2—C1—C6—C5	−0.9 (8)	C7—C12—O14—Ba	−42.3 (9)
N1—C1—C6—C5	−179.8 (5)	C11—C12—O14—Ba	140.1 (5)
C12—C7—C8—C9	0.0 (8)	C2—O7—Ba—O17	105.6 (4)
N4—C7—C8—C9	−179.1 (5)	C2—O7—Ba—O15	−85.0 (4)
C7—C8—C9—C10	1.5 (8)	C2—O7—Ba—O14	7.9 (4)
C7—C8—C9—N5	−179.1 (5)	C2—O7—Ba—O16	155.6 (4)
C8—C9—C10—C11	−2.2 (8)	C2—O7—Ba—O18	49.6 (6)
N5—C9—C10—C11	178.4 (5)	C2—O7—Ba—O8	−44.7 (4)
C9—C10—C11—C12	1.5 (9)	C2—O7—Ba—O1	65.4 (4)
C9—C10—C11—N6	−178.9 (5)	C2—O7—Ba—O11^iii^	174.8 (4)
C8—C7—C12—O14	−178.6 (5)	C2—O7—Ba—O5^i^	−138.9 (4)
N4—C7—C12—O14	0.5 (8)	C12—O14—Ba—O7	−25.0 (6)
C8—C7—C12—C11	−0.7 (7)	C12—O14—Ba—O17	−130.9 (6)
N4—C7—C12—C11	178.4 (4)	C12—O14—Ba—O15	99.6 (6)
C10—C11—C12—O14	177.9 (6)	C12—O14—Ba—O16	−74.3 (6)
N6—C11—C12—O14	−1.8 (8)	C12—O14—Ba—O18	169.9 (6)
C10—C11—C12—C7	0.0 (8)	C12—O14—Ba—O8	39.5 (6)
N6—C11—C12—C7	−179.7 (5)	C12—O14—Ba—O1	−76.1 (6)
C6—C1—N1—O1	−156.2 (5)	C12—O14—Ba—O11^iii^	169.6 (5)
C2—C1—N1—O1	24.8 (7)	C12—O14—Ba—O5^i^	25.7 (7)
C6—C1—N1—O2	24.6 (7)	N4—O8—Ba—O7	85.4 (8)
C2—C1—N1—O2	−154.4 (5)	N4—O8—Ba—O17	1.1 (10)
C6—C5—N2—O3	−176.0 (6)	N4—O8—Ba—O15	−129.7 (8)
C4—C5—N2—O3	2.4 (8)	N4—O8—Ba—O14	−18.9 (7)
C6—C5—N2—O4	2.7 (8)	N4—O8—Ba—O16	110.0 (8)
C4—C5—N2—O4	−178.9 (6)	N4—O8—Ba—O18	−71.1 (8)
C4—C3—N3—O6	−144.6 (5)	N4—O8—Ba—O1	34.8 (8)
C2—C3—N3—O6	35.4 (7)	N4—O8—Ba—O11^iii^	−144.0 (7)
C4—C3—N3—O5	33.1 (7)	N4—O8—Ba—O5^i^	152.1 (8)
C2—C3—N3—O5	−146.9 (5)	N1—O1—Ba—O7	−45.6 (5)
C8—C7—N4—O8	−164.9 (5)	N1—O1—Ba—O17	178.3 (6)
C12—C7—N4—O8	15.9 (7)	N1—O1—Ba—O15	52.7 (7)
C8—C7—N4—O9	15.2 (7)	N1—O1—Ba—O14	63.4 (5)
C12—C7—N4—O9	−164.0 (5)	N1—O1—Ba—O16	−115.5 (5)
C8—C9—N5—O10	−1.7 (8)	N1—O1—Ba—O18	128.4 (5)
C10—C9—N5—O10	177.7 (6)	N1—O1—Ba—O8	15.6 (5)
C8—C9—N5—O11	177.6 (5)	N1—O1—Ba—O11^iii^	−165.4 (5)
C10—C9—N5—O11	−3.0 (8)	N1—O1—Ba—O5^i^	−70.9 (5)

Symmetry codes: (i) −*x*+2, −*y*+2, −*z*; (ii) *x*+1, *y*, *z*; (iii) *x*−1, *y*, *z*.

### Hydrogen-bond geometry (Å, °)

**Table:**

*D*—H···*A*	*D*—H	H···*A*	*D*···*A*	*D*—H···*A*
O15—H15A···O7^iv^	0.85 (4)	2.20 (4)	2.939 (6)	145 (6)
O15—H15A···O6^iv^	0.85 (4)	2.33 (4)	3.030 (7)	140 (5)
O15—H15B···O9^v^	0.85 (4)	2.103 (6)	2.953 (6)	179 (7)
O16—H16A···O4^iii^	0.85 (3)	2.08 (4)	2.806 (6)	142 (5)
O16—H16B···O15^vi^	0.85 (6)	2.11 (3)	2.906 (7)	156 (6)
O17—H17A···O12^vii^	0.85 (4)	2.45 (3)	3.258 (8)	158 (6)
O17—H17B···O18^vii^	0.85 (5)	1.969 (17)	2.805 (7)	168 (7)
O18—H18A···O16^iv^	0.85 (4)	2.04 (3)	2.822 (7)	153 (7)
O18—H18B···O1^viii^	0.85 (5)	2.47 (3)	3.241 (7)	151 (6)

Symmetry codes: (iv) *x*, *y*−1, *z*; (v) −*x*+2, −*y*+1, −*z*; (iii) *x*−1, *y*, *z*; (vi) *x*, *y*+1, *z*; (vii) −*x*+2, *y*+1/2, −*z*+1/2; (viii) −*x*+2, *y*−1/2, −*z*+1/2.
